# DiAlert: a prevention program for overweight first degree relatives of type 2 diabetes patients: results of a pilot study to test feasibility and acceptability

**DOI:** 10.1186/1745-6215-13-178

**Published:** 2012-09-27

**Authors:** Wieke H Heideman, Maartje de Wit, Barend JC Middelkoop, Vera Nierkens, Karien Stronks, Arnoud P Verhoeff, Frank J Snoek

**Affiliations:** 1 Department of Medical Psychology, The EMGO Institute for Health and Care Research, VU University Medical Center, Amsterdam, The Netherlands; 2 Department of Public Health and Primary Care, Leiden University Medical Centre, Leiden, The Netherlands; 3 Department of Public Health, Academic Medical Centre, University of Amsterdam, Amsterdam, The Netherlands; 4 Department of Epidemiology, Documentation and Health Promotion, Amsterdam Municipal Health Service, Amsterdam, The Netherlands; 5 Department of Sociology and Anthropology, University of Amsterdam, Amsterdam, The Netherlands

## Abstract

**Background:**

Prevalence of type 2 diabetes mellitus is increasing due to lifestyle changes, particularly affecting those genetically at risk. We developed DiAlert as a targeted group-based intervention aimed to promote intrinsic motivation and action planning for lifestyle changes and weight loss in first degree relatives of patients with type 2 diabetes mellitus.

The main objective of the pilot of the DiAlert intervention was to assess fidelity, feasibility and acceptability prior to starting the randomized controlled trial.

**Methods:**

Individuals with a family history of type 2 diabetes mellitus were self-identified and screened for eligibility. DiAlert consists of two group sessions. Feasibility, fidelity, acceptability and self-reported perceptions and behavioral determinants were evaluated in a pre-post study using questionnaires and observations. Determinants of behavior change were analyzed using paired-samples t tests and Wilcoxon signed rank tests.

**Results:**

DiAlert was delivered to two groups of first degree relatives of patients with type 2 diabetes mellitus (N = 9 and N = 12). Feasibility and fidelity were confirmed. Overall, the DiAlert group sessions were positively evaluated (8.0 on a scale of 1 to 10) by participants. The intervention did not impact perceived susceptibility or worry about personal diabetes risk. Action planning with regard to changing diet and physical activity increased.

**Conclusions:**

DiAlert proved feasible and was well-accepted by participants. Positive trends in action planning indicate increased likelihood of actual behavior change following DiAlert. Testing the effectiveness in a randomized controlled trial is imperative.

**Trial registration:**

Netherlands National Trial Register (NTR): NTR2036

## Background

The increasing prevalence of type 2 diabetes mellitus (T2DM) is associated with high rates of morbidity and mortality and is a growing public health burden [1]. T2DM is a multifactorial disease and reflects an interaction between genetic susceptibility and lifestyle. Large trials have convincingly shown that lifestyle interventions with the aim to lose 5% to 7% body weight are associated with significant health benefits and more than 50% reduction of risk for developing T2DM [2,3]. However, these interventions are highly intensive and offered in experimental settings, and translating these findings to primary care with limited resources has proven to be challenging [4-6]. Less intensive diabetes prevention programs targeted at high-risk individuals could increase the uptake and effectiveness, particularly if such programs are linked to primary care and lifestyle services in the community. We developed DiAlert as a lifestyle education program in primary health care settings, in response to the need for applicable, effective interventions for overweight people with a positive family history of T2DM, with the aim to prevent T2DM [7]. DiAlert is explicitly designed as a short, structured education program, focusing on key determinants of health behavior change, with a focus on promoting risk awareness and motivation for lifestyle changes. In this paper, we report on the development and first experiences with DiAlert in a pilot study.

DiAlert is a theory-based group intervention, consisting of two interactive group sessions and a participant’s manual. The aim of DiAlert is to reduce diabetes risk by means of weight loss. To this purpose, personal risk awareness and intrinsic motivation are enhanced, along with personal goal setting and action planning for lifestyle changes. The program follows the format of the Diabetes Education for Self-Management in Ongoing and Newly Diagnosed (DESMOND) program [8] that was adapted to PRo-active Interdisciplinary Self-Management (PRISMA) in the Netherlands [9]. The development of the DiAlert intervention included a review of existing lifestyle interventions for people with a positive family history of T2DM [10] and expert meetings.

DiAlert was informed by the Health Action Process Approach (HAPA) framework [11], a model based on social learning theories with strong empirical support. HAPA identifies three key determinants of initial change: risk perception, self-efficacy and outcome expectancies, which feed into intentions (motivation) that then need to be translated into action plans to achieve and maintain actual behavior change. DiAlert targets all key determinants of the framework to help participants create a personal action plan to lose weight. In this pilot study, we seek to examine whether the DiAlert intervention has any effect on these determinants.

Following the Medical Research Council framework for complex interventions [12], which provides guidance on the development, evaluation and implementation of complex interventions in health care, we carried out an in-depth development fidelity and feasibility phase, to ensure that the design of the DiAlert intervention was appropriate for the target group and setting. The DiAlert intervention sought to help participants to lose weight to decrease T2DM risk. Formative evaluations were obtained to identify factors in the intervention that work well or are in need of improvement. Prior to commencing the main randomized controlled trial (RCT) to test the effectiveness of the intervention, we set out to accomplish four objectives. The first three of these were to asses the fidelity (were intervention modules delivered as intended?), feasibility (was delivery of the intervention feasible in terms of time, group size, amount of sessions, etc.?) and acceptability (did participants, observers and trainer evaluate positively the content and group format positively?) of the intervention sessions, by looking at the process of delivering the intervention. Fourth, we assessed pre-post changes following the DiAlert intervention on the specified determinants of behavior change.

## Methods

The pilot study was conducted at the outpatient clinic of the VU University Medical Center in Amsterdam in November 2010. The Medical Ethics Committee of the VU University Medical Center approved the study protocol. Inclusion criteria for participation were being a first degree relative of a patient with T2DM; age between 29 and 55 years; and being overweight (body mass index of ≥25 kg/m^2^).

### Recruitment

We aimed to recruit sufficient participants (approximately 20) for two groups to be able to test the intervention twice. Approximately 250 flyers and information leaflets were posted in the hospital building and outpatient clinic of the VU University Medical Center in Amsterdam plus an advertisement run in a local newspaper in October 2010. In addition, announcements were posted on the project website ( http://www.dialert.nl) and the website of VU university medical center ( http://www.vumc.nl). To trigger attention, all recruitment materials included the sentence ‘Does diabetes run in your family?’ along with information about the pilot study and inclusion criteria. Participants were allowed to bring relatives who met inclusion criteria to participate in the pilot study. Participants received a small incentive and reimbursement of travel expenses.

### Intervention

DiAlert is offered in two sessions of 150 minutes, and delivered over a period of two weeks by a trained health educator (WH), henceforth referred to as the trainer. The trainer was instructed according to a standardized training program for the PRISMA (Dutch DESMOND) program [9].

Participants are encouraged to explore possibilities and resources for prevention in a positive atmosphere and using a constructive didactic approach. In view of the fact that participants are simply overweight and symptom free, with a family history of T2DM, we assume an interest in the program but perhaps not a strong readiness for change, as one might expect in those who are medically ill. Therefore, DiAlert puts emphasis on promoting risk awareness and intrinsic motivation for changing lifestyle, while avoiding inducing psychological distress. The empowerment philosophy supports the educational process to develop awareness and autonomy to effectively assume responsibility for their decisions in relation to lifestyle behavior. In line with this philosophy, the trainer is guiding rather than teaching. Following the format of PRISMA, a group size of 8 to 10 participants was considered to be ideal with ample opportunity for participants to interact with the trainer and the other group members.

**Figure 1:**
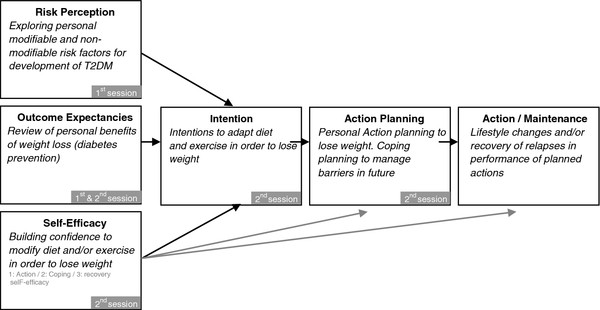
Behavioral change determinants of the Health Action Process Approach framework, applied for the DiAlert intervention.

The development of DiAlert was guided by the HAPA framework (Figure 1). HAPA builds on social cognitive theory, distinguishing two stages of behavior change: motivation, and action and maintenance. The basic assumption underlying HAPA is that motivation is a necessary condition for behavior change, but that goal-setting and action planning are required for the change to actually occur. Throughout the process of behavior change, feelings of self-efficacy play a key role.

In the first session, adequate risk perception is promoted by providing information and discussion of modifiable and non-modifiable risk factors for T2DM. To increase positive outcome expectancies for weight loss and physical activity, the trainer introduces the topic of combined insulin resistance and loss of beta cell function as the underlying pathophysiology of diabetes, using simple analogies and schemes. The benefits of weight loss and physical activity are discussed in that context. In relation to weight loss, energy balance (intake and expenditure) is discussed. At the end of the first session, participants are asked to record nutrition and physical activity behavior in a diary on two separate days during the week in between the two group sessions. Self-monitoring of their health behaviors will help participants set personalized and realistic goals.

In the second session, self-efficacy beliefs, strategies to lose weight, coping strategies, goal setting and action planning are addressed.

Patients are provided with a written curriculum highlighting the key points and including the diary and action planning sheets. During group sessions, worries, discussions and clarifications put forward by the participants and trainer are written down on large flip-over sheets, visible for all participants, that stay there for the whole program. During the modules about healthy food choices, wrappings and containers of commonly used products are displayed and discussed. A more detailed description of the intervention development and outline is given elsewhere [7].

### Measures

Participants were asked to fill out a questionnaire twice, one week before the first group session and four weeks later (that is, one week after the last group session). See Tables 1 and 2 for an overview of the measures and times of data collection. In addition, the flip-over sheets produced during the sessions gave information about main interests, questions and worries of the participants.

**Table 1:** Analysis of fidelity, feasibility and acceptability of the DiAlert intervention

**Category of measurement**	**Instrument and stage (by whom)**	**Topics measured**	**Scale**	**Results**
**Fidelity measures**	Checklists	Coverage of the role of the trainer and the participants	Checklist coverage: yes/no tick box	Observations with checklists showed that all modules were delivered. The role of the trainer and the objectives for participants were covered.
	-during the intervention sessions(Observers)			
	Evaluations	Engagement of participants	Observations	Engagement was high, demonstrated by active questioning by participants, active participation at the calorie games, most participants completing the homework assignment and attendance in both group sessions.
	-after each group session (Observers and Trainer)			
				Attrition was low: one participant was absent at the second session.
		Empowerment philosophy	Observations	The trainer supports the empowerment philosophy during both sessions, see checklist for items of empowerment.
	Quotes of participants written down on flip-over sheets	Do relatives of T2DM patients have:	Quotes of participants	Participants have worries about:
				1. Relatives (for example ‘worries about my mom/dad/ children’
				2. Own health: (for example, ‘I’m afraid of getting diabetes myself’; ‘I think I’m too young to get it [diabetes]!’
	-during the first session (Trainer)	1. worries?		
		2. questions?	Quotes of participants	Main themes of burning questions:
				1. Diabetes causality and its relation to lifestyle (for example, ‘What is the primary cause of T2DM? Does stress affect development of T2DM’, ‘How important is eating healthy food, and what is considered to be healthy?’)
				2. Questions about diabetes treatment and complications (for example, ‘Why do some people receive pills and others insulin treatment?’, ‘How can someone prevent getting polyneuropathy?’).
		3. interests in relation diabetes prevention?	Quotes of participants	Categories of reasons to participate:
				1. Risk awareness and worry (for example ‘My risk of getting diabetes is high’)
				2. Information seeking (for example, ‘How are lifestyle and diabetes risk related?’)
				3. Motivation (for example, ‘Stimulates me to improve my exercise behavior’).
	Questionnaire	- perceptions of worry [13]	1 = totally not worried	No significant changes for worry about personal risk and personal control of developing T2DM, for example:
	-at baseline			
			7 = very worried	
				Indicate your feelings when thinking about chance of getting diabetes: baseline 5.0 ± 1.6; follow-up 5.0 ± 1.6; *P* = 0.92)
	−4 weeks follow-up (Participant)			
		- personal control [13]	1 = totally disagree	No significant changes for personal control of developing T2DM, for example:
			5 = totally agree	
				I think I have little influence on getting T2DM: baseline 2.5 ± 1.1; follow-up 1.9 ± 1.0; *P* = 0.08
				I can reduce my risk of getting diabetes: baseline 4.3 ± 0.7; follow-up 4.3 ± 1.2; *P* = 0.92
				I think I have little control over my own health: baseline 1.8 ± 0.7; follow-up 1.8 ± 0.7; *P* = 0.85
		- perceived consequences of T2DM [13]	1 = totally disagree	Significant increase of perceived consequences of getting T2DM, for example:
			5 = totally agree	
				Major implications for life: baseline 4.2 ± 0.8; follow-up 4.5 ± 0.7; *P* = 0.04
				Major financial implications^a^: baseline 2.9 ± 1.1; follow-up 3.4 ± 1.0; *P* <0.01
**Feasibility measures**	Questionnaire	- which recruitment strategies were appropriate / How did participants knew about the study?	Multiple choice including 1 open-ended option.	Recruitment through flyers and advertisements n = 14 (66%), announcement on internet n = 3 (14.4%) and via a relative n = 3 (14.4%)
	-at baseline (Participant)			
	Observations	- time, duration of the modules/sessions	Minutes per module reported on checklist	All modules were delivered within 2 × 150 minutes; duration of modules deviated sometimes from planned time.
	-during the intervention sessions (Observer)			
	Questionnaire	- length of sessions was good:	1 = totally disagree	90% of the participants evaluated the length of the sessions ‘good’ score ≥3
	-follow-up 4 weeks (Participant)			
			4 = totally agree	
	Evaluation form	- group size	Multiple choice: too small, just right, too large	All participants evaluated the group size ‘just right’
	-at the end of second session (Participant)			
**Acceptability measures**	Evaluation form	- generic grade for total intervention: (mean ± SD)	1 (lowest grade)	8.0 ± 1.0
	-at the end of second session (Participant)			
			10 (highest grade)	
		- usefulness of the separate modules (mean ± SD)	1 = very useful	Introduction 1.5 ±0.5; Risk factors 1.3 ±0.5; Development of diabetes 1.3 ±0.6; Homework 1.8 ±0.9; Information about physical activity 1.4 ±0.5; Information about diet 1.5 ±0.8; Action plan 1.7 ±0.8; Questions 1.5 ±0.7
			5 = totally not useful	
	Questionnaire	- participants manual: instructive and clear	1 = totally disagree	Instructive 3.4 ±0.5; clear 3.4 ±0.5
	-follow-up 4 weeks (Participant)			
			4 = totally agree	
		- action plan: managed to make one and useful	1 = totally disagree	Managed to make an action plan 2.8 ±0.5; useful to create a personal action plan 3.1 ±0.6
			4 = totally agree	
		(mean ± SD)		

*SD* standard deviation; *T2DM* type 2 diabetes mellitus.

**Table 2:** Mean baseline and follow-up values for analysis of determinants of behavior change

**Determinant of behavioral change (HAPA)**	**Domain and instrument**	**Questions (scale)**	**Baseline**	**Follow-up**
			**N = 17**	**N = 16**
**Risk perception**	1. Causal beliefs: (Revised Illness Perception Questionnaire) [14]	*Indicate the extent to which you believe that a given cause could be a cause of diabetes (1 = definitely not; 5 = definitely)*		
		- Heredity	4.3 ±0.7	4.4 ±0.9
		- Aging	4.2 ±0.8	4.1 ±1.0
		- Lifestyle (smoking, alcohol use, lack of physical activity and nutrition habits)^a,b^	4.0 ±0.6	4.0 ±1.0
		- Stress or worry	3.3 ±1.3	3.2 ±1.4
		- Country of origin	3.2 ±1.6	4.1 ±1.0^e^
	2. Comparative risk: adopted from Claassen *et al*. [13]	W*hat is the chance of you getting diabetes compared with an average man/woman your age? (1 = a lot lower; 7 = a lot higher)*	5.4 ±1.0	4.7 ±1.3
	3. Risk estimation [13]	*How big is the chance of you getting diabetes within the next 5 years? ( 1 = very small; 7 = very big )*	4.7 ±1.5	4.7 ±1.3
**Outcome expectancies**	For healthy diet and increasing physical activity: adopted from Schwarzer *et al*. [15]	1. Diet: *If I eat healthy foods: I feel healthy/I will lose weight/I will look better/I feel relaxed (1 = totally disagree; 5 = totally agree) (sum score 4 to 20)*^*a,b*^	16.0 ±2.5	15.9 ±1.7
		2. Physical activity: *If I exercise more: I feel healthy/I will lose weight/I will look better (1 = totally disagree; 5 = totally agree) (sum score 3 to 15)*^*a,c*^	12.2 ±1.4	12.5 ±1.4
**Self-efficacy**	For healthy diet and physical activity: adopted from Schwarzer *et al*. [16]	1. Diet: *I am confident that I can eat healthy food - even if I: need a long time to develop the necessary routines/try several times until it works/have to rethink my entire way of nutrition/do not receive a great deal of support from others when making my first attempts/have to make a detailed plan (1 = very unconfident; 4 = very confident) (sum score 4 to 20)*^b^	13.8 ±3.2	13.8 ±3.1
		2. Physical activity: *I can manage to carry out my exercise intentions even when I: have worries and problems/feel depressed/feel tense/am tired/am busy. (1 = very unconfident; 4 = very confident) (sum score 4 to 20)*^*b*^	12.0 ±3.3	12.6 ±4.0
**Intentions**	For healthy diet, physical activity losing weight [15]	*In the next three months I’m going to: (1 = totally disagree; 5 = totally agree)*		
		1. eat healthy	3.7 ±0.9	3.6 ±1.0
		2. exercise more	3.7 ±0.9	3.8 ±0.9
		3. lose weight	3.9 ±0.7	3.7 ±1.0
**Planning**	For healthy diet and physical activity [15]	1. Diet: *I have concrete plans… what/how to change nutrition habits/what to do in difficult situations in order to stick to my intentions.*		
		*(1 = totally disagree to 4 = tot ally agree) (sum score 3 to 12)*^*a,b*^		
		2. Physical activity: *I have concrete plans when/where/how/how many times/with whom I’m going to exercise/what to do in difficult situations in order*		
		*to stick to my intentions (1 = totally disagree; 4 = totally agree) (sum score 6 to 24)*^*d*^		

All determinants were assessed with questionnaires at baseline and 4-weeks follow-up. Performed analyses were Wilcoxon signed rank tests, and t-test (^a^) in case of normal distributions. ^b^Cronbach’s alpha of sum scores ≥0.8, ^c^Cronbach’s alpha of sum scores of 3 items 0.67; ^d^Cronbach’s alpha of sum scores >0.9, ^e^*P* <0.05. HAPA: Health Action Process Approach.

### Participant characteristics

Characteristics of the study population were assessed by self-report, including sociodemographics, family history of diabetes in first and second degree relatives, body weight and lifestyle behavior.

### Fidelity measures

To assess whether the intervention was delivered consistently with the underlying theory and philosophy and to what extent the intervention was delivered as planned, the sessions were observed and findings recorded on a checklist. The checklist was created based on the objectives of the program and trainers instructions. Two independent observers attended the group sessions and were instructed to check whether all modules were delivered and all objectives for participants were covered (see Additional file 1); to report on the engagement of participants by looking at interactions between trainer and participants and among participants; and to observe whether the sessions were delivered in a constructive, empowering atmosphere (that is, the trainer listens and is respectful and empathetic to all participants).

The worries, questions and reasons for participation discussed in the first session provided insight into the extent to which the goals of the program matched those of the participants. In addition, we measured worry about diabetes risk, feelings of personal control and perceived consequences by means of questionnaires at baseline and follow-up [13]. These outcomes could also show whether DiAlert had any adverse effects on these perceptions.

### Feasibility measures

Information on feasibility is essential before embarking on a RCT in a larger sample. For the aim of this pilot, we explored recruitment strategies, by asking participants how they knew about the intervention and why they were participating in the intervention. Length of the modules was timed with a stopwatch by the observers and recorded on the checklist and we examined whether all information could be delivered in two sessions of 150 minutes. Group size was informed by PRISMA and evaluated to confirm acceptability and feasibility (evaluation form: too small, just right, too large), observations (were all participants involved in the intervention?) and experiences of the trainer (was it feasible to deliver the intervention as intended with this number of participants?). Length of the sessions was assessed in the follow-up questionnaire.

### Acceptability measures

Participants’ views and experiences with the DiAlert program were assessed using a short evaluation form at the end of the second group session and with the questionnaire at follow-up.

The evaluation form asked participants to give an overall grade between 1 and 10 for the whole intervention and to rate usefulness of each module of the intervention and the homework assignment on a five-point Likert scale (see Table 1).

At follow-up, we assessed whether participants would recommend the DiAlert program to others. Participants were asked to respond on two statements about the manual: ‘In my opinion the information in the manual of DiAlert is… clear/instructive’ and to evaluate the action plan ‘I managed to create personal goals’ and ‘I think it is useful to create a personal action plan’.

### Determinants of behavioral change

In line with the HAPA framework (Figure 1) determinants of behavioral change were made operational by questions at baseline and follow-up, derived from existing measures. Risk perception for diabetes was assessed on three different domains: causal beliefs, using a validated questionnaire [14]; comparative risk; and risk estimation using questionnaires adopted from former studies in the field of family risk information [13]. To assess the other determinants we adopted questionnaires from Schwarzer *et al*.: perceived self-efficacy for healthy eating and physical activity was assessed by 10 questions [16]. Outcome expectancies for a healthy diet and increasing physical activity were measured with eight questions adopted from Schwarzer *et al*. [15]. Intentions and action planning to change health behaviors were examined, asking participants whether they plan to consciously eat healthier, exercise more or lose weight and if they have formulated a detailed action plan (what, when, how) for changing diet and physical activity [15]. See Table 2 for exact wording of the questions.

### Data analysis

Fidelity, feasibility and acceptability measures were analyzed descriptively, using data from the questionnaire at follow-up, the evaluation forms and observers’ checklists. Contributions of participants written down on the flip-over sheets and free text evaluations from the follow-up questionnaires we used to illustrate opinions of participants. Pre-post comparisons of changes in the determinants of behavioral change were conducted in overweight participants using paired-samples t tests or Wilcoxon signed rank tests. A *P*-value of <0.05 was considered to be statistically significant in all analyses. Statistical analyses were performed using SPSS 15.0 (SPSS Inc., Chicago, IL, USA).

## Results

### Participant characteristics

In total, 22 people signed up for the DiAlert pilot study and participated in two different groups, 10 and 12 participants respectively. Twenty participants had a first degree relative with T2DM, one participant had no first degree relatives but did have a number of second degree relatives with diabetes and was allowed to participate. Of the participants with a first degree relative with T2DM, two had a sibling with diabetes; all others reported parental family history of T2DM. One participant appeared not to have any relative with T2DM, and was therefore removed from the analyses, leaving 21 participants for baseline analyses. Characteristics of the participants are described in Table 3. The majority of participants was female (86%) with a mean age of 47.9 ±9.7 years. Most participants were from Dutch descent (N = 15), others reported Surinamese, Moroccan, Hindustani, Indian, Polish and Chinese ethnicities. All participants spoke Dutch fluently. Mean self-reported body mass index was 29 ±6.3 kg/m^2^. More than half of the participants (N = 13) had attempted to lose weight in the past five years with a mean number of 5.9 ± 4.5 attempts.

**Table 3:** Baseline characteristics of participants

**Characteristic (N = 21)**	
**Age (years)**	47.9 ±9.7
**Female**	18 (85.7%)
**Positive family history**	
A first degree relative only	20 (95.2%)
A second degree relative only	1 (4.8%)
Both first and second degree relatives	6 (28.6)
**Weight (kg)**	81.1 ±17.5
**Body mass index (kg/m**^**2**^**)**	29.0 ±6.3
Normal (18 to 25)	4 (19%)
Overweight (25 to 30)	10(47.6%)
Obese (≥30)	7 (33.3%)
**Reported elevated blood sugar in the past (yes)**	7 (33.3%)
**Earlier attempts weight loss attempts (yes)**	13 (61.9%)
Mean number of attempts	5.9 ±4.5
**Current smoker (yes)**	6 (28.5%)
**Education**^**a**^	
Lower	10 (47.6%)
Middle	4 (19.0%)
Higher	7 (33.3%)
**Employed (yes)**	12 (57.1%)
**Marital state - living with partner**	11 (52.4%)
**Self reported ethnicity**	
Dutch	15 (71.4%)
Other	6 (28.6%)

Values are presented in number of participants (%) or mean ± SD. ^a^Lower education = primary education or lower general secondary education; middle = intermediate vocational education or high school; high = higher vocational.

One participant dropped out after the first session, due to family circumstances.

### Fidelity of the intervention

All topics of the intervention were covered in the two sessions as planned, in both groups, and all materials developed were used. All participants received the participant manual and took it home. In general, engagement of the participants was high in both groups - all participants were actively involved in both sessions. Participants were particularly engaged in the module discussing calories of displayed food products. Observers confirmed that the intervention was delivered in an empowering atmosphere.

As shown in Table 1, examination of the flip-over sheets showed that family risk information was an important topic of discussion and participants expressed concerns about their own risk of developing diabetes. Also, concerns were expressed about relatives, in most cases the parents who were having problems controlling their diabetes. In addition, the risk of diabetes in own offspring was raised in one group. Two main themes emerged from the listed ‘burning questions’ at the beginning of the sessions. First, questions about causes of diabetes and its relation to lifestyle, and secondly, both groups raised questions about T2DM treatment and complications, see Table 1 for examples of quotes from participants.

No significant changes were found for the items on personal control and worry about personal risk, indicating that the intervention had no effects on these determinants. Perceived consequences of T2DM slightly increased at follow-up and participants more often disagreed with the statement: ‘I think I have little influence on getting type 2 diabetes’.

### Feasibility

Recruitment resulted in a sufficient number of participants for two groups within a relatively short period of three weeks. Twenty-five people showed interest in participating in the pilot study and contacted the research team by email or telephone. Three people decided not to participate after receiving more detailed information. Both younger and older individuals showed interest in the DiAlert intervention, therefore we decided to include participants from 25 to 65 years old. Although the DiAlert intervention was targeted at overweight relatives, four participants had a normal body mass index (<25 kg/m^2^), and were included because the main aim of this pilot was to evaluate the process and feasibility of the group sessions. Most participants signed up after reading about DiAlert in flyers and the newspaper advertisement (63.7%). Main reasons for participating in the DiAlert pilot study were: ‘prevention of T2DM’ (N = 9) and ‘learning about the personal risk of diabetes due to a family history’ (N = 5). In addition, the three motives for joining the program that were mentioned at the beginning of the first session were risk awareness and worry, information seeking and motivation to change behavior.

The feasibility of the group sessions was confirmed in terms of duration of the modules and group size. All modules were delivered within the scheduled 150 minutes. Some modules exceeded the planned time with a maximum of 10 minutes, while other modules took less time.

The group size was evaluated as ‘just right’ by participants in both groups. Most participants (90%) stated that the duration of the interventions was good.

At the end of the sessions, observers and trainer confirmed that delivery of DiAlert is feasible in line with the empowerment philosophy and theoretical framework. In both groups, all participants were able to formulate goals and create a personal action plan to lose weight. The homework assignment was completed by almost all participants (N = 20) in between the two sessions.

### Acceptability

Following the underlining empowerment philosophy, participants seemed to be encouraged to play an active role in the intervention, and came up with examples and questions to be answered during the sessions. High engagement of participants was noticed in both groups, especially sections with activities, where all participants were involved and came to the table to discuss calories and food choices together. After one week of follow-up, participants gave an overall mark of 8.0 ±1.0 on a scale from 1 (lowest) to 10 (highest). All participants would recommend the DiAlert program to others. Overall, evaluation of the usefulness of the intervention modules showed a mean score of 1.5 ±0.4 (scale, 1 = very useful to 5 = totally not useful). The module ‘development of diabetes’ got the highest rating 1.3 ±0.6. In this module the development of diabetes is discussed with participants using a drawing to explain insulin resistance, loss of beta cell function and the positive effects of body weight loss and physical activity. Evaluation of the information in the manual and its clarity was good, 3.4 ±0.5 (scale, 1 = totally disagree to 4 = totally agree).

### Determinants of behavioral change

Because the main objective of DiAlert is weight loss, post intervention analyses of the determinants of behavior change were performed for the overweight participants only (N = 16). Analyses of baseline questionnaire scores showed that participants were aware of the main risks for developing T2DM at baseline, with mean scores of >4 (scale, 1 = definitely not to 5 = definitely) on the items nutrition, heredity, aging and lack of physical activity (Table 2). Not surprisingly, we found a relatively high baseline score for heredity (4.3 ±0.7). Participants perceived their risk (comparative risk and risk estimation) somewhat higher than average. Sum scores for outcome expectancies for a healthy diet and for physical activity were 16.0 ±2.5 (scale 1 to 20) and 12.2 ±1.4 (scale 1 to 15), respectively. This suggests that participants had quite positive outcome expectancies already for eating healthy foods and doing exercise.

Small non-significant increases in self-efficacy and outcome expectancies for diet and physical activity appeared at follow-up. Furthermore, at follow-up, all participants stated they were more aware of their risk, 65% said they ate more healthily due to DiAlert, and 40% improved their physical activity. Causal beliefs for the item ‘country of origin’ increased significantly (*P* = 0.04), probably explained by discussion of heredity and the relation with genetic predisposition in the first session of the intervention.

With regard to action planning, significant positive changes were seen for both diet (*P* = 0.008) and physical activity (*P* <0.001). This means that the participants were more able to formulate concrete plans to change their dietary habits and physical activity pattern, including addressing coping plans to anticipate difficult situations in the future.

## Discussion

The main aim of this pilot study was to evaluate fidelity, feasibility and acceptability of the DiAlert intervention before testing efficacy in a RCT. We took the opportunity to describe the development of a complex intervention and to share our lessons learned of developing an intervention in this specific target group at risk for T2DM. In our opinion, confirmation of content and delivery of the intervention is very important before conducting the intervention in a RCT setting.

The pilot study showed that the new lifestyle education program DiAlert is attractive and feasible for relatives of patients with T2DM. Evaluation of fidelity showed no deficiencies and the intervention was delivered as theorized. All modules were delivered in time and the intervention was highly appreciated by participants. DiAlert helped participants to create personal action plans aimed at changing dietary habits and/or increasing physical activity to lose weight. This is an important finding since action planning is an important mediator of successful health behavior change [17]. In all group sessions a positive atmosphere was noted, despite the topic of health risks and the need for lasting lifestyle changes. Moreover, our pilot study gave no indication that the risk information provided in the two group sessions resulted in fatalism or extreme worries. This result together with previous studies suggests that targeted diabetes risk information for relatives of patients with T2DM can increase engagement in risk-reducing behaviors [18-20] without causing psychological harm [18-21]. An important finding was that some participants expressed concerns and worry about their family members developing T2DM and complications, and were for that reason more interested in learning about diabetes.

Most participants in this pilot study were overweight women who were sedentary and not meeting recommendations for a healthy diet or physical activity. We attracted both lower and higher educated participants for this pilot, which adds to the external validity of our findings. The results from the pilot seem to indicate that heterogeneity with regard to educational level, health profile (previous health warnings, overweight) and cultural background fits well with the program.

In this pilot, not all participants were overweight. However, we were able to test the DiAlert intervention program on its fidelity, because in this pilot phase we focused mainly on applicability of the intervention, delivery of the intervention and appreciation of participants.

### Lessons learned from this pilot

Some issues relating to the conduct and management of the future RCT have been raised by this pilot study. First, interest in the topic was confirmed based on the finding that recruitment efforts proved effective to reach a sufficient number of eligible participants. However, mainly women were reached; therefore, in the RCT, we should take into account possible strategies to include both men and women and from a broad range of socioeconomic classes. In the RCT, we will apply a mixed recruitment strategy, involving general practitioners and diabetes specialists together with use of media and brochures to recruit participants with a positive family history of T2DM directly. As result of a direct recruitment approach (through general practitioners) we may expect participants with lower perception of risk, less positive outcome expectancies and lower self-efficacy for lifestyle changes. Another issue in relation to recruitment was that, although the DiAlert study was initially aimed at relatives 29 to 55 years of age, younger and older people showed interest and were enrolled. Therefore, the inclusion criteria for the upcoming RCT will be changed to 25 to 65 years of age to certify validity of the intervention. We plan to deliver a culturally-sensitive version of the DiAlert intervention to relatives of Turkish origin living in the Netherlands. The intervention will be pretested in this target group before we conduct the RCT in this group.

Second, discussion of risk information did not increase worries about personal risk. However, we should keep in mind that participants did express concerns and worries about their own family members with T2DM. Some participants clearly were in need of information on diabetes and its management with regards to their relatives rather themselves. In the RCT, the focus of DiAlert should stay on prevention and risk of developing diabetes due to family history, rather than discussing problems that may occur in their relatives with T2DM in the future.

From this pilot, we learned that no changes to the intervention modules are anticipated before embedding DiAlert in a RCT.

## Conclusions

DiAlert is a structured educational intervention based on principles of self-management that has been shown to be feasible and of interest to people genetically predisposed to T2DM. We demonstrated that participants were willing and able to formulate action plans after two group sessions. The DiAlert intervention was deliberately designed as a short and interactive intervention, to enhance the attractiveness of the program for people at risk who are overweight and not yet medically ill. Finding the balance between attractiveness and high reach on the one hand and efficacy on the other is challenging, but preliminary results are promising. The group education approach could contribute to the implementation of primary prevention programs in primary care to educate persons at risk in a cost-efficient way. Further investigation of DiAlert will involve a RCT, looking at both behavioral and metabolic outcomes.

## Competing interests

The authors declare that they have no competing interests.

## Authors’ contributions

WH coordinated and delivered the pilot intervention and drafted the manuscript; MdW helped to draft and revise the manuscript; BM, VN, KS and AV participated in the design of the study and revised the manuscript. FS developed the study and revised the manuscript. All authors read and approved the final manuscript.

## Supplementary Material

Additional file 1 Checklist DiAlert intervention sessions. Click here for file
