# Supplementary material for: DiAlert: a prevention program for overweight first degree relatives of type 2 diabetes patients: results of a pilot study to test feasibility and acceptability
Source: Trials. 2012 Sep 27;13:178. doi: 10.1186/1745-6215-13-178 (PMC3543339; doi:10.1186/1745-6215-13-178)
Supplement: Additional file 1 — Checklist DiAlert intervention sessions. [file 1745-6215-13-178-S1.doc]

### Additional file 1 – checklist DiAlert intervention sessions

| **First session** | | | | |
| --- | --- | --- | --- | --- |
|  | **Duration** |  | **Role of trainer** | **Objectives for participants** |
| **A.** | 5  … MIN | **INTRODUCTION** | - Welcome to all participants and introduction of trainer and observers. - Stating aims of the intervention - Today’s modules | - Know when pause / coffee / tea / snacks are - Friendly and relaxed atmosphere - Respect to each other |
| **B.** | 20  … MIN | **PARTICIPANT TOPICS** | - Facilitate participants to share experiences/worries about diabetes - List questions of participants - Everyone in the group gets the opportunity to tell his/her story | - Getting to know each other - Sharing views and experiences regarding type 2 diabetes in the family - Know reasons for participation - Participants feel free ask questions |
| **C.** | 35  … MIN | **VIEW ON PERSONAL RISK FACTORS** | - Risk factors for / causes of T2DM - Modifiable risk factors (overweight, diet, lack of physical activity, stress or worries) - Non-modifiable risk factors (genetics, age, ethnicity) - Discuss personal risk profile - Benefits of weight loss | - Know what factors influence the risk of diabetes - Explores personal modifiable risk factors |
| **D.** | 30  … MIN | **HOW TO PREVENT T2DM?** | - Discuss insulin resistance, loss of beta cell function and positive effects of weight loss and physical activity. - Differences between type 1 and type 2 diabetes - Diet: introduction of calories - Summarize information - Trainer asks whether participant agree or doubt the information | - Knowing what goes wrong in the body in the case of type 2 diabetes - Know the term 'insulin resistance' and its relation to overweight and lack of exercise - Understand that there is decreased insulin production caused by exhaustion of the pancreas - Knowing positive effects of weight loss and exercise - Get familiar with the term "calories" |
| **E.** | 15  … MIN | **HOMEWORK ASSIGNMENT** | - Ask participants to fill out the diary for two days - Trainer explains that the diary is for personal use | - Knowing that the diary is personal. Personal details won’t be discussed in the group session |
| **F.** | 5  … MIN | **CONCLUSION** | - Positive take home message: Confirm trust that diabetes can be prevented - Next session: topics, place, time and location - Importance of second session - Summary | - Acquaintance with the topics of next session - Experience with options to prevent diabetes |
| **Second session** | | | | |
| **G.** | 10  … MIN | **REFLECTIONS** | - Participants get the opportunity to express concerns and ask new questions - Encourage participants to address overweight as a modifiable risk factor for diabetes - Summary : opportunities for diabetes prevention - Appoint Today’s Topics | - Identify questions he/she want to address - Acquainted with 1^st^ sessions information - Understands overweight affects the development of diabetes |
| **H.** | 40  … MIN | **TAKING CONTROL: NUTRITION AND EXERCISE BALANCE** | - Ask about experiences with the diary. - Promote active participation in the calorie game - Encourages participants to reflect on options to lose weight - Balance between energy intake and expenditure - Summary | - Can describe methods to reduce weight, by means of energy balance - The ability to use food labels to determine how many calories a given product contains - Being aware that the relationship between energy intake and expenditure must be balanced |
| **I.** | 45  … MIN | **PERSONAL ACTIONPLAN** | - Stimulate participants to choose one personal risk factor they want to change encourage participants to seek which self-management behaviors they could change - Identify success factors - Help participants create a SMART (specific, measurable, assignable, realistic, timely) goal  - What, how, when, who etc - Help participants to specify the formulated goal:  - How confident are you that it will work? - What barriers are there? - Summary | - Create at least 1 objective to reduce weight - Knowing the potential barriers for achieving their goal - Have confidence to achieve goal |
| **J.** | 15  … MIN | **BURNING QUESTIONS** | - Make sure that participants know the answers to their questions or that they know where and from whom they can get these answers | - Getting answers to all questions - Knows where to get answers to remaining questions |
| **K.** | 15  … MIN | **CONCLUSIONS** | - Conclusions - Thanks for participating |  |
| *To support the empowerment philosophy in general during both sessions,*  The trainer:   - Makes no judgments of statements made by participants - Uses open questions to ensure the participant’s understanding - Gives each participant the opportunity to explore/ express views the topic discussed - Enables participants to reflect on topics discussed - Adapts the pace of the modules to of learning of participants | | | | |
